# Clinical Significance and Functional Insights of Tesmin in Hepatocellular Carcinoma

**DOI:** 10.1155/2024/3058875

**Published:** 2024-01-19

**Authors:** Lijun Cao, Lin Zhang, Siyu Liu, Xue Wang

**Affiliations:** Department of Infection, The Fourth Affiliated Hospital of Harbin Medical University, Harbin City, Heilongjiang Province, China

## Abstract

**Background:**

Tesmin, a 60 kDa protein encoded by the metallothionein-like 5 (MTL5) gene, plays a vital role in spermatogenesis and oogenesis. Recent research has unveiled its potential involvement in malignancies, although its impact on HCC remains poorly understood.

**Methods:**

In this study, we sought to elucidate the clinical significance of tesmin in HCC patients. We investigated the relationship between tesmin expression and the prognosis of individuals with hepatocellular carcinoma (HCC), as well as its potential role in tumor proliferation and invasion. Immunohistochemistry (IHC) was employed to assess the expression of tesmin in HCC tissues. Chi-square tests were conducted to analyze the correlation between tesmin expression and various clinicopathological features among HCC patients. For survival analysis, we employed the Kaplan–Meier method and conducted Cox regression analyses. To investigate the functional role of tesmin, we utilized shRNA constructs for transfection-mediated knockdown. Proliferation was assessed using the CCK-8 assay, and invasive capability was determined through Matrigel Transwell assays.

**Results:**

IHC results indicated that tesmin expression was prominently observed in cancerous tissue. Notably, we observed a significant association between tesmin expression and tumor stage and invasion in HCC patients from both our medical center and TCGA dataset. Survival analysis further revealed that tesmin expression emerged as an independent prognostic factor for overall survival among individuals with HCC. Furthermore, cellular experiments demonstrated that knockdown of tesmin led to decreased proliferation and invasion of HCC cells.

**Conclusions:**

Our findings suggest that tesmin may serve as a novel prognostic marker for HCC, highlighting its potential as a target for further research into HCC treatment. Additionally, the functional experiments support the notion that tesmin may participate in promoting the proliferation and invasion of HCC cells, warranting further investigations into its mechanistic involvement in HCC progression.

## 1. Introduction

Liver cancer, primarily hepatocellular carcinoma (HCC), represents a significant global health challenge due to the high incidence and mortality rates [1]. HCC arises from the hepatocytes of the liver and is often diagnosed at an advanced stage, limiting the effectiveness of available therapeutic interventions [2]. Understanding molecular mechanisms underlying HCC development and progression is critical for improving diagnosis, prognosis, as well as therapeutic strategies for this devastating disease [3].

One protein of emerging interest in the context of hepatocellular carcinoma is tesmin, a 60 kDa protein encoded by the metallothionein-like 5 (MTL5) gene [4]. Initially recognized for its pivotal roles in spermatogenesis and oogenesis [5, 6], recent investigations have shed light on tesmin's potential involvement in malignancies beyond its reproductive functions. A previous study reported that MTL5 rs12365708, a missense mutation (Cys to Arg) of MTL5 may contribute to susceptibility to childhood B-cell acute lymphoblastic leukemia (ALL). According to Natalie's data, maternal MTL5 rs12365708 is associated with childhood ALL in both their cohort (RR = 2.62, 95% CI = 1.61–4.27) and meta-analysis (pooled RR = 2.27, 95% CI = 1.48–3.50) [7]. In addition, Sanada et al. reported that MTL5 gene was a prognostic factor for patients with lung adenocarcinoma [8]. These reports have indicated that tesmin may influence tumor progression, suggesting its potential as a therapeutic target and prognostic marker in multiple cancer types. However, the specific relationship between tesmin and hepatocellular carcinoma remains relatively unexplored.

This study aims to bridge this knowledge gap by exploring the clinical significance of tesmin in patients with hepatocellular carcinoma. We seek to elucidate the association between tesmin expression and the prognosis of HCC patients, as well as its role in tumor proliferation and invasion. Through immunohistochemistry and functional experiments, we assess tesmin's expression in HCC tissues and its impact on tumor behavior. This research may provide critical insights into tesmin as a potential novel prognostic marker and therapeutic target for hepatocellular carcinoma, offering new avenues for understanding and managing this deadly disease.

## 2. Methods

### 2.1. Ethical Approval

The present study obtained ethical approval from the Fourth Affiliated Hospital of Harbin Medical University, and all procedures were conducted in accordance with the Declaration of Helsinki and relevant ethical guidelines. Written informed consent was obtained from all patients included in the study.

### 2.2. Study Population

A total of 128 HCC patients who underwent R0 resection and survived for more than 3 months postsurgery were enrolled from the Fourth Affiliated Hospital of the Harbin Medical University for this retrospective cohort study. The follow-up period ranged from 3 to 72 months, with a median follow-up time of 20 months. Clinical and pathological information of these patients was collected, including age, sex, hepatitis B virus (HBV) infection status, alpha-fetoprotein (AFP) level, lesion number, tumor size, tumor side, pathological grade, histological differentiation, resection margin, portal vein invasion, and TNM stage.

### 2.3. Immunohistochemistry (IHC)

Immunohistochemistry was performed to assess the expression of tesmin in HCC tissues. Briefly, formalin-fixed, paraffin-embedded HCC tissue sections were deparaffinized, rehydrated, and subjected to antigen retrieval. After blocking with serum, the sections were incubated with anti-tesmin antibodies. Subsequently, the sections were treated with secondary antibodies, and diaminobenzidine was used for visualization. Staining intensity and percentage of positive cells were evaluated for each sample [9].

### 2.4. Kaplan–Meier Survival Analysis

Overall survival (OS) was analyzed using the Kaplan–Meier method. Survival curves were generated, and log-rank tests were used to assess the significance of differences in survival rates between subgroups [10]. Median survival time, 3-year OS, and 5-year OS rates were calculated.

### 2.5. Cox Regression Analysis

Multivariate analysis was performed using Cox regression analysis to identify independent prognostic factors for HCC. Hazard ratios (HRs) and their corresponding 95% confidence intervals (CIs) were calculated. Variables included in the analysis were lesion number, tumor size, tumor side, pathological grade, portal vein invasion, TNM stage, and tesmin expression. Variables with *p* values <0.05 were considered statistically significant.

### 2.6. Cell Culture and In Vitro Experiments

HCC cell lines Hep 3B2.1-7 and HepG2 were used for in vitro experiments. Knockdown of tesmin was achieved using two distinct short hairpin RNAs (shRNAs), with scrambled shRNA serving as a control. Western blot experiments were conducted to validate the efficiency of tesmin knockdown. Cell proliferation was assessed using the CCK-8 assay, and invasion capacity was evaluated by the Matrigel Transwell assay.

### 2.7. Online Dataset

We also retrieved data from The Cancer Genome Atlas (TCGA) database to investigate the correlation between MTL5 gene expression levels and various clinicopathological features of HCC patients in the TCGA cohort. The dataset included comprehensive clinical and gene expression information of HCC patients. To assess the relationship between MTL5 gene expression and clinical characteristics, we analyzed the following parameters: TNM stage, histological grade, AFP level, and vascular invasion. The MTL5 gene expression levels were correlated with these clinical characteristics using statistical methods.

### 2.8. Analysis of Immune Infiltration

Furthermore, we explored the potential associations between MTL5 gene expression and immune infiltration in HCC using the TCGA dataset. Specifically, we investigated whether MTL5 expression correlated with immune activation. To evaluate these associations, we conducted bioinformatic analyses using the ESTIMATE (Estimation of STromal and Immune cells in MAlignant Tumor tissues using Expression data) algorithm applied to the TCGA HCC dataset. ESTIMATE is a tool utilized to predict tumor purity and the infiltration of stromal and immune cells within tumor tissues based on gene expression data. This algorithm is rooted in single-sample gene set enrichment analysis (ssGSEA). It primarily generates three scores: stromal score (indicating the presence of stromal cells in tumor tissue), immune score (indicating the infiltration of immune cells in tumor tissue), and estimate score (indicating tumor purity) [11, 12].

### 2.9. Statistical Analysis

Statistical analyses were performed using SPSS version 22.0. The relationship between tesmin expression and clinicopathological features was assessed using the Chi-square test. Cox regression analysis was conducted to identify independent prognostic factors for HCC. A *p* value <0.05 was considered statistically significant. All *p* values reported were two tailed.

## 3. Results

### 3.1. Online Data Collection and Analysis

**Figure 1:**
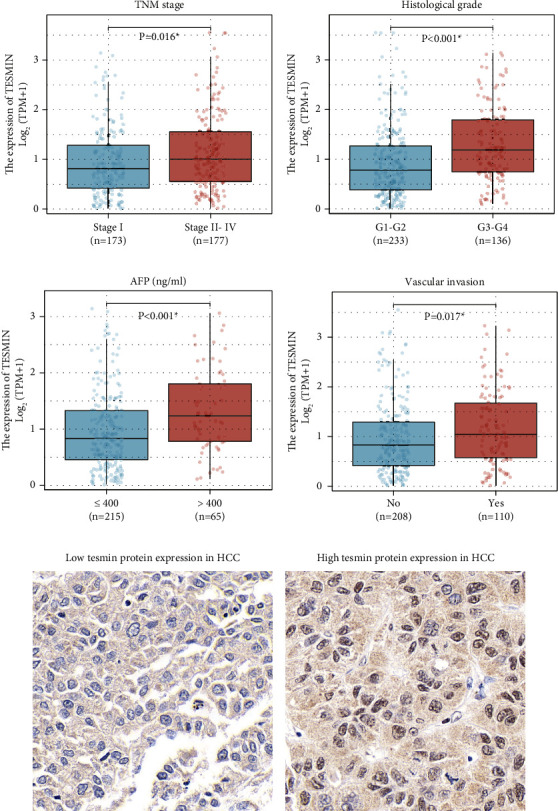
MTL5 gene level and tesmin protein expression in HCC specimens. (a) Correlations between MTL5 gene level and TNM stage of HCC patients in the TCGA cohort. (b) Correlations between MTL5 gene level and histological grade of HCC patients in the TCGA cohort. (c) Correlations between MTL5 gene level and serum AFP level of HCC patients in the TCGA cohort. (d) Correlations between MTL5 gene level and vascular invasion of HCC patients in the TCGA cohort. (e) Immunohistochemistry staining depicting low protein expression of tesmin in HCC specimens. (f) Immunohistochemistry staining showing high protein expression of tesmin in HCC specimens.

Firstly, we assessed MTL5 gene expression levels in HCC specimens from the TCGA cohort according to the TPM (transcript per million). Our analysis revealed significant correlations between MTL5 level and key clinical parameters using the Mann–Whitney *U* test. For example, MTL5 levels were notably elevated in HCC patients with advanced TNM stages (Figure 1(a)), indicating a potential link to disease progression. Additionally, higher MTL5 expression was associated with poorer histological differentiation (Figure 1(b)) and elevated serum AFP levels (Figure 1(c)), suggesting a role in tumor grade and clinical markers. Furthermore, positive vascular invasion was significantly linked to increased MTL5 expression, hinting at its involvement in tumor invasion and metastasis (Figure 1(d)). These findings collectively highlight MTL5's potential as a valuable biomarker in HCC prognosis and progression.

### 3.2. Enrolled Patients' Information from Our Medical Center

In this study, we retrospectively investigated a cohort of 128 HCC patients in our hospital who had all undergone R0 resection and survived for a minimum of 3 months postsurgery. At the conclusion of the follow-up period, 79 patients had unfortunately succumbed to the disease, while 49 individuals were censored. The follow-up duration ranged from 3 to 72 months, with a median follow-up time of 20 months. Patient demographics (Table 1) revealed that 76 patients were younger than 50 years, while 52 were aged 50 years or older. In terms of gender distribution, 33 patients were female, and 95 were male. Hepatitis B virus (HBV) infection status was categorized as negative in 41 cases and positive in 87 cases, reflecting the diversity of this patient cohort.

Furthermore, our analysis considered key clinical characteristics. Serum AFP levels were segmented into less than 400 ng/mL (44 cases) and equal to or greater than 400 ng/mL (84 cases). The number of lesions in the liver was classified as single in 90 cases and multiple in 38 cases. Tumor size was divided into less than 5.0 cm (76 cases) and equal to or greater than 5.0 cm (52 cases). Tumor location was described as unilateral in 113 cases and bilateral in 15 cases. Pathological grade showed a distribution of 91 cases with Grade 1-2 and 37 cases with Grade 3. Histological differentiation was evenly split between well/moderate (64 cases) and poor/undifferentiated (64 cases). The assessment of resection margins revealed 44 cases with margins equal to or greater than 1.0 cm and 84 cases with margins less than 1.0 cm. Lastly, portal vein invasion was categorized as negative in 96 cases and positive in 32 cases. The TNM stage exhibited 57 cases in TNM I-II and 71 cases in TNM III-IV.

### 3.3. Tesmin Protein Expression and Its Correlation with Patients' Characteristics

We next conducted IHC analyses for the cases enrolled above to assess the protein expression level of tesmin. IHC results indicated that 54 cases with low tesmin expression (Figure 1(e)), while the other 74 cases with high tesmin protein expression levels (Figure 1(f)).

Table 1 provides a comprehensive overview of tesmin protein expression within our cohort of 128 enrolled HCC patients and its association with various clinical variables using the Chi-square test. Notably, tumor size, portal vein invasion, and TNM stage displayed statistically significant correlations with tesmin expression. Tumor size less than 5.0 cm was linked with high tesmin expression, in contrast to larger tumors (≥5.0 cm) where low tesmin expression prevailed. Moreover, tesmin expression was significantly elevated in patients with portal vein invasion and those in TNM stages III-IV, suggesting its potential as a valuable prognostic marker in assessing disease progression and outcomes. Conversely, tesmin expression did not exhibit significant associations with patient age, gender, HBV infection status, AFP levels, lesion number, pathological grade, histological differentiation, or resection margin. The correlations between patients' characteristics and tesmin protein expression in our cohort are consistent with their correlations with MTL5 gene level in the TCGA cohort. These findings underscore tesmin's potential utility as a biomarker specifically linked to certain critical aspects of HCC, such as tumor size, invasiveness, and disease stage, offering valuable insights into its clinical relevance.

### 3.4. Survival Analyses Highlight the Prognostic Significance of Tesmin in HCC

Table 2 presents the results of Kaplan–Meier survival analyses for our cohort of 128 HCC patients, offering insights into the prognostic significance of various clinical variables. Age, gender, and HBV infection status did not exhibit statistically significant associations with overall survival (OS). While age displayed a trend, with younger patients showing a 3-year OS rate of 37.2% and older patients at 46.7%, the difference did not reach significance (*P*=0.317). Gender revealed a notable trend, with females displaying a 3-year OS rate of 26.4% compared to 47.0% for males (*P*=0.065). HBV infection status also did not significantly affect survival, with negative patients at a 3-year OS rate of 43.2% and positive patients at 36.7% (*P*=0.466).

**Figure 2:**
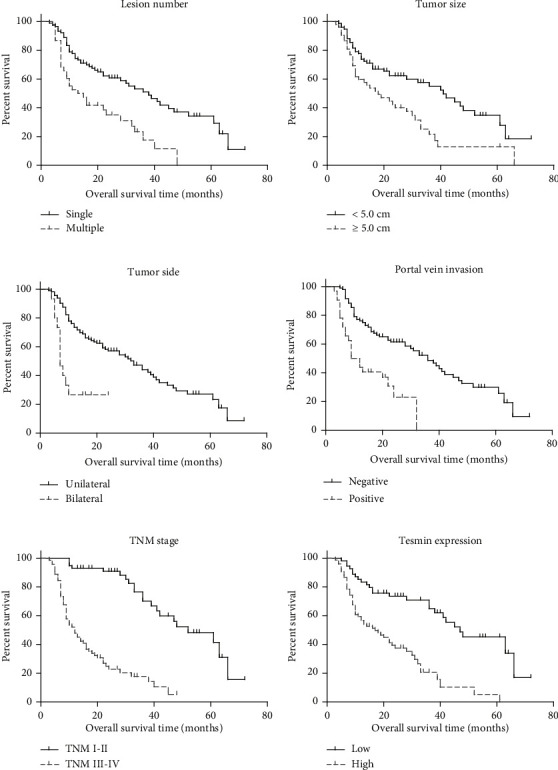
Overall survival analyses. (a) Kaplan–Meier survival curves based on lesion number for enrolled HCC patients. (b) Kaplan–Meier survival curves based on tumor size for enrolled HCC patients. (c) Kaplan–Meier survival curves based on tumor laterality for enrolled HCC patients. (d) Kaplan–Meier survival curves based on portal vein invasion for enrolled HCC patients. (e) Kaplan–Meier survival curves based on TNM stage for enrolled HCC patients. (f) Kaplan–Meier survival curves based on tesmin expression level for enrolled HCC patients. Log-rank tests were used to compare the survival data.

In contrast, several other factors strongly influenced survival outcomes. Lesion number and tumor size were impactful, as patients with a single lesion exhibited a 3-year OS rate of 51.0%, while those with multiple lesions had a considerably lower rate of 17.5% (*P*=0.416, Figure 2(a)). Similarly, patients with tumors smaller than 5.0 cm had a 3-year OS rate of 55.0%, whereas those with larger tumors equal to or greater than 5.0 cm had a rate of 21.6% (*P*=0.002, Figure 2(b)). Tumor side also played a vital role in survival, with unilateral tumor patients experiencing a 3-year OS rate of 44.0%, while bilateral tumor patients had a rate of 26.7% (*P* < 0.001, Figure 2(c)). Furthermore, portal vein invasion and TNM stage had substantial impacts on survival, as patients without portal vein invasion exhibited a 3-year OS rate of 48.7%, while those with portal vein invasion had a 3-year OS rate of 0% (*P* < 0.001, Figure 2(d)). TNM I-II patients demonstrated a 3-year OS rate of 70.1%, whereas TNM III-IV patients had a significantly lower rate of 17.7% (*P* < 0.001, Figure 2(e)). Most notably, tesmin expression emerged as a robust prognostic factor, with patients displaying low tesmin expression enjoying a substantially higher 3-year OS rate of 64.9% compared to those with high tesmin expression, who had a notably lower 3-year OS rate of 20.7% (*P* < 0.001, Figure 2(f)). These findings highlight high tesmin expression as an unfavorable prognostic factor in hepatocellular carcinoma, underscoring its potential clinical significance.

Table 3 summarizes the results of multivariate analysis using Cox regression analysis, providing insights into the independent prognostic significance of various clinical variables in HCC patients. Notably, tumor size (≥5.0 cm vs. <5.0 cm) and tumor side (bilateral vs. unilateral) emerged as robust independent adverse prognostic factors, with hazard ratios (HRs) of 2.797 (95% CI: 1.645–4.754) and 4.703 (95% CI: 2.254–9.814), respectively, both associated with highly significant *p* values (<0.001). Additionally, TNM stage (III/IV vs. I-II) demonstrated strong independent prognostic significance, with an HR of 6.755 (95% CI: 3.516–12.978, *p*  <  0.001), underlining its critical role in overall survival prediction. Furthermore, high tesmin expression (vs. low) was identified as an independent unfavorable prognostic factor, with an HR of 1.939 (95% CI: 1.054–3.566) and a *p* value of 0.033, underscoring its relevance as a prognostic marker in HCC. Conversely, lesion number, pathological grade, and portal vein invasion did not exhibit independent prognostic significance in this analysis. These results emphasize the importance of tumor-related factors, TNM stage, and tesmin expression in independently predicting the prognosis of HCC patients, offering valuable insights for risk assessment and clinical decision-making.

### 3.5. Functional Involvement of Tesmin in HCC Progression

**Figure 3:**
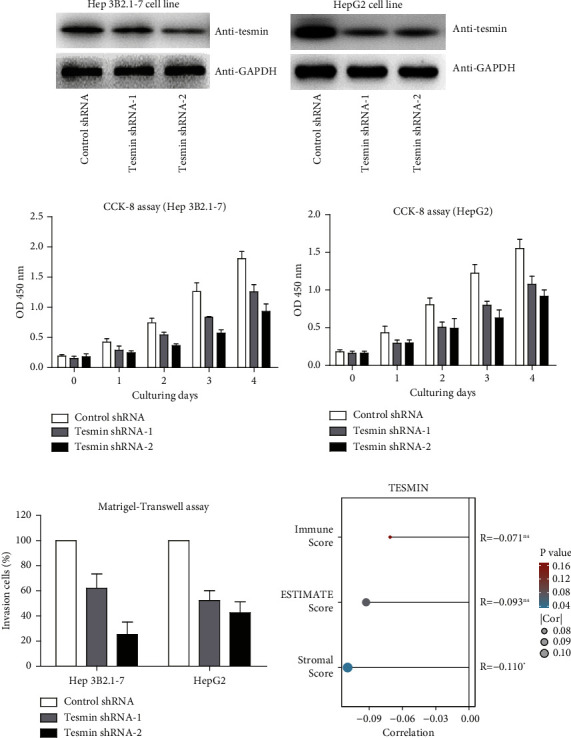
Cellular effects of tesmin knockdown in HCC cells. (a, b) Western blot experiments assessing the efficiency of tesmin knockdown using two shRNAs, with scrambled shRNA as a control. These experiments were conducted in two HCC cell lines, Hep 3B2.1-7 and HepG2. (c, d) The CCK-8 assay reveals reduced cell proliferation viability in both Hep 3B2.1-7 and HepG2 cell lines following tesmin knockdown compared to control cells treated with scrambled shRNA. (e) Matrigel Transwell assay results indicate that tesmin knockdown by shRNAs impair the invasion capacities of both Hep 3B2.1-7 and HepG2 cell lines. (f) Analysis of MTL5 gene levels suggests potential correlations with immune activation, particularly the stromal score, in HCC according to the TCGA dataset.

To further explore the potential role of tesmin in HCC progression, we conducted cellular experiments using two HCC cell lines, Hep 3B2.1-7 and HepG2. Western blot (WB) experiments were conducted to assess the efficacy of tesmin knockdown using two shRNAs, with scrambled shRNA serving as a control (Figures 3(a and b)). Immunoblotting data indicate that the shRNAs effectively lowered tesmin levels in these HCC cells. Turning to functional outcomes, CCK-8 assays reveal that tesmin knockdown resulted in a notable reduction in cell proliferation viability in both Hep 3B2.1-7 and HepG2 cell lines when compared to control cells treated with scrambled shRNA, underscoring the role of tesmin in promoting cell proliferation in the context of HCC (Figure 3(c and d)).

Moreover, as reflected by the Matrigel Transwell assay, tesmin knockdown by shRNAs significantly impaired the invasion capacities of both Hep 3B2.1-7 and HepG2 cell lines (Figure 3(e)). This functional effect highlights tesmin's involvement in promoting invasive behavior in HCC cells. Finally, we delve into the potential correlations between MTL5 gene levels and immune activation in HCC based on the TCGA dataset (Figure 3(f)). This analysis suggests intriguing links between tesmin expression and the immune microenvironment in hepatocellular carcinoma, providing further insights into the multifaceted role of tesmin in HCC.

## 4. Discussion

This study investigated the clinical significance of tesmin in HCC and its potential role in tumor proliferation and invasion. The major findings of this study revealed that high tesmin expression was significantly associated with advanced tumor stage, poorer differentiation, higher serum AFP levels, and positive vascular invasion. Moreover, tesmin emerged as an independent adverse prognostic factor for overall survival in HCC patients. In functional experiments, tesmin knockdown led to reduced cell proliferation and impaired invasion capacities in HCC cell lines. These findings collectively highlight the potential of tesmin as a novel prognostic marker and therapeutic target in HCC.

The association between tesmin expression and unfavorable clinicopathological features in HCC is in line with recent reports suggesting the involvement of tesmin in malignancies. Our results resonate with studies demonstrating elevated tesmin expression in other cancer types, including ovarian cancer and lung cancer. In cervical cancer, tesmin has been linked to cancer progression and metastasis [13], aligning with our observations of tesmin's impact on HCC invasion. Furthermore, the relationship between high tesmin expression and poorer differentiation underscores the potential role of tesmin in tumor aggressiveness and dedifferentiation, a phenomenon also observed in other malignancies. For example, tesmin was reported to be higher expressed in non-small cell lung cancer (NSCLC) and cervical tissues and correlated with patients' survival [13, 14]. Moreover, another research group reported higher levels in NSCLC cells than lung fibroblast control cells from both mRNA and protein levels [15].

The finding that high tesmin expression is an independent adverse prognostic factor for overall survival in HCC patients is of paramount significance. This aligns with previous studies identifying tesmin as a prognostic marker in other cancers such as cervical cancer [13], emphasizing its potential as a universal prognostic indicator across various malignancies. These results warrant further exploration of tesmin as a therapeutic target in HCC, as its downregulation impaired both proliferation and invasion capacities in HCC cell lines. This aligns with previous research in cervical cancer, where tesmin knockdown inhibited cell proliferation and migration, supporting its potential as a therapeutic target. Similarly, silencing of tesmin induced a decreased cell number in the G2 phase of NSCLC cells, indicating its role in modulating cell cycle [14]. Consistent with its proliferation potential, another study also reported its positive correlation with Ki-67 expression level based on IHC results, further emphasized its role in promoting tumor growth [15].

Nevertheless, this study has several limitations. Firstly, the retrospective nature of the cohort introduces inherent biases and may limit the generalizability of the findings. Secondly, the functional experiments were conducted in vitro, and the precise molecular mechanisms underlying tesmin's effects on HCC remain to be elucidated. Additionally, the potential relationship between tesmin and the immune microenvironment, as suggested by our analysis of MTL5 gene levels, warrants further investigation. Lastly, the study does not encompass a comprehensive analysis of treatment modalities and their potential interactions with tesmin expression.

## 5. Conclusions

In conclusion, this study elucidates the clinical significance of tesmin in HCC, with high tesmin expression serving as an independent predictor of poor prognosis. The functional experiments underscore its role in tumor proliferation and invasion. These findings open avenues for further research into tesmin as a potential prognostic marker and therapeutic target in HCC, with the ultimate goal of improving patient outcomes. However, given the limitations of this study, future prospective studies and mechanistic investigations are warranted to validate and expand upon these findings.

## Figures and Tables

**Table 1:** Expression of tesmin protein in enrolled patients.

Variables	Cases	Tesmin expression		*P* value
	(*n* = 128)	Low (*n* = 54)	High (*n* = 74)	
Age				
<50 yrs	76	31	45	0.699
≥50 yrs	52	23	29	
Sex				
Female	33	11	22	0.232
Male	95	43	52	
HBV infection				
Negative	41	21	20	0.155
Positive	87	33	54	
AFP level				
<400 ng/mL	44	21	23	0.358
≥400 ng/mL	84	33	51	
Lesion number				
Single	90	40	50	0.426
Multiple	38	14	24	
Tumor size				
<5.0 cm	76	47	29	<0.001^*∗*^
≥5.0 cm	52	7	45	
Tumor side				
Unilateral	113	48	65	0.855
Bilateral	15	6	9	
Pathological grade				
Grade 1-2	91	42	49	0.154
Grade 3	37	12	25	
Histological differentiation				
Well/moderate	64	22	43	0.073
Poor/undifferentiated	64	32	32	
Resection margin				
≥1.0 cm	44	17	27	0.556
<1.0 cm	84	37	47	
Portal vein invasion				
Negative	96	49	47	<0.001^*∗*^
Positive	32	5	27	
TNM stage				
TNM I-II	57	32	25	0.004^*∗*^
TNM III-IV	71	22	49	

*Note*. ^*∗*^Statistically significant by Pearson Chi-square test or Fisher's exact test.

**Table 2:** Overall survival by Kaplan–Meier analyses.

Variables	Patients	Overall survival months		*P* value
	(*n* = 128)	3-year OS (%)	Mean ± S.D.	
Age				
<50 yrs	76	37.2	30.7 ± 2.8	0.317
≥50 yrs	52	46.7	35.9 ± 4.0	
Sex				
Female	33	26.4	25.2 ± 2.7	0.065
Male	95	47.0	35.9 ± 2.9	
HBV infection				
Negative	41	43.2	34.5 ± 4.0	0.466
Positive	87	36.7	31.7 ± 2.8	
AFP level				
<400 ng/mL	44	57.4	37.3 ± 3.7	0.146
≥400 ng/mL	84	33.6	30.5 ± 2.8	
Lesion number				
Single	90	51.0	37.9 ± 2.9	<0.001^*∗*^
Multiple	38	17.5	20.2 ± 2.6	
Tumor size				
<5.0 cm	76	55.0	39.1 ± 3.2	0.002^*∗*^
≥5.0 cm	52	21.6	24.5 ± 3.1	
Tumor side				
Unilateral	113	44.0	34.8 ± 2.5	<0.001^*∗*^
Bilateral	15	26.7	11.4 ± 2.0	
Pathological grade				
Grade 1-2	91	46.5	35.6 ± 2.8	0.060
Grade 3	37	27.9	26.8 ± 4.2	
Histological differentiation				
Well/moderate	64	41.0	31.8 ± 3.1	0.658
Poor/undifferentiated	64	41.1	33.8 ± 3.3	
Resection margin				
≥1.0 cm	44	45.6	36.4 ± 4.2	0.406
<1.0 cm	84	39.2	30.7 ± 2.6	
Portal vein invasion				
Negative	96	48.7	36.9 ± 2.6	<0.001^*∗*^
Positive	32	0	15.6 ± 2.0	
TNM stage				
TNM I-II	57	70.1	49.9 ± 3.1	<0.001^*∗*^
TNM III-IV	71	17.7	17.7 ± 1.7	
Tesmin expression				
Low	54	64.9	45.1 ± 3.6	<0.001^*∗*^
High	74	20.7	22.1 ± 2.2	

*Note*. ^*∗*^Statistically significant by log-rank test.

**Table 3:** Multivariate analysis by Cox regression analysis.

Variables	Hazard ratio	95% CI	*P* value
Lesion number (multiple vs. single)	1.443	0.846–2.460	0.178
Tumor size (≥5.0 cm vs. <5.0 cm)	2.797	1.645–4.754	<0.001^*∗*^
Tumor side (bilateral vs. unilateral)	4.703	2.254–9.814	<0.001^*∗*^
Pathological grade (grade 3 vs. grade 1-2)	0.864	0.517–1.441	0.574
Portal vein invasion (positive vs. negative)	1.485	0.793–2.779	0.216
TNM stage (III/IV vs. I-II)	6.755	3.516–12.978	<0.001^*∗*^
Tesmin expression (high vs. low)	1.939	1.054–3.566	0.033^*∗*^

*Note*. ^*∗*^Statistically significant by Cox regression analysis.

## Data Availability

The data used to support the findings of this study are available from the corresponding author upon reasonable request.
